# Toxic Epidermal Necrolysis Following the Ingestion of Paracetamol in a Pediatric Patient: A Case Report

**DOI:** 10.7759/cureus.48216

**Published:** 2023-11-03

**Authors:** Aditya Jain, Revat J Meshram

**Affiliations:** 1 Department of Pediatrics, Jawaharlal Nehru Medical College, Datta Meghe Institute of Medical Sciences School of Epidemiology and Public Health, Wardha, IND

**Keywords:** toxic epidermal necrolysis, steven johnson syndrome, paracetamol, chicken pox, adverse drug reaction

## Abstract

Paracetamol is considered to be a relatively safe drug, even in the pediatric age group, at the recommended doses. Here we present a case of a six-year-old male presenting with symptoms and signs of Steven Johnson syndrome/toxic epidermal necrosis (SJS/TEN) following the ingestion of paracetamol. Steven Johnson syndrome/toxic epidermal necrosis is a potentially life-threatening dermatological emergency requiring intensive treatment. The patient was initially misdiagnosed as a case of chickenpox and was administered paracetamol. However, upon attending a tertiary care facility, he was diagnosed with TEN and treated with immunosuppressants. He recovered fully without any complications and was discharged within a week.

## Introduction

Steven Johnson syndrome (SJS) is a rare immunoreaction following exposure to certain pharmacological agents or infections [1,2]. The normal immune-mediated reaction is exaggerated, and there is overstimulation of cytotoxic T cells and natural killer (NK) cells and an ensuing cascade of cell signaling and release of inflammatory mediators [3]. These, in turn, also cause apoptotic changes in the peripheral tissues, leading to blistering, skin necrosis, and sloughing [4]. Additionally, necroptosis also contributes to programmed cell death via the interaction of annexin A1 from monocytes and the formyl peptide receptor 1 [5]. When the involvement of total body surface area is less than 10%, it is termed SJS; more than 30% is toxic epidermal necrosis (TEN); and 10% to 30% is a mixed syndrome (SJS/TEN) [6]. Diagnosis can be made by typical dermatological manifestations with a history of prior exposure to the offending agent. A biopsy is rarely required to confirm the diagnosis. Treatment is mainly provided by meticulous supportive care and immunosuppressive agents.

## Case presentation

A six-year-old male child was brought by the parents to the emergency department with the chief complaint of erosive lesions all over the body. On detailed history-taking, it was noted that the patient had developed complaints of fever eight days prior with a body ache without any history of cough, cold, or loose stools. There was also no history of similar complaints in any contacts. He was treated with a paracetamol suspension. The fever was relieved after three doses. However, within two days, he started developing multiple well-defined erosions with an erythematous base associated with itching. It started with the torso and gradually became generalized. The next day, there were oral ulcerations along with peeling of the skin.

Subsequently, after two days, the patient was taken to a nearby government hospital, where he was diagnosed with a varicella zoster infection and treated with an acyclovir injection, calamine lotion, and paracetamol tablets. There was no reported relief, and the patient was referred to our hospital upon receiving no relief.

**Figure 1:**
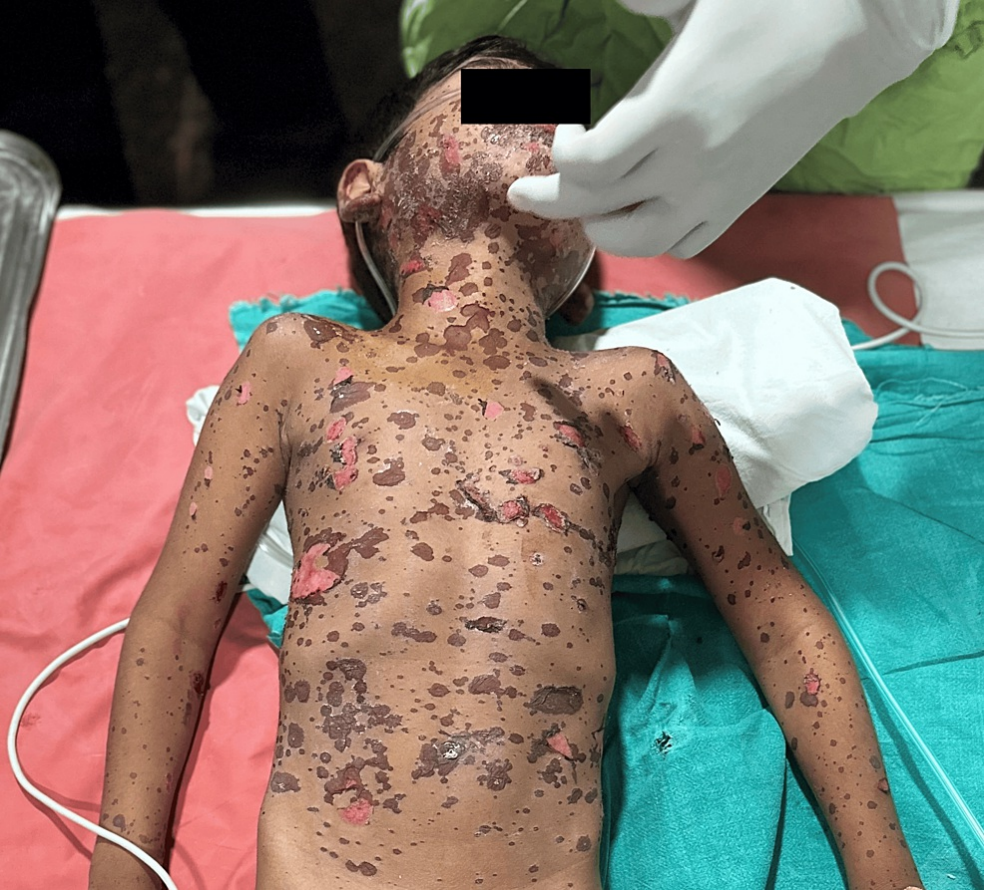
Lesions on the front side of the body

**Figure 2:**
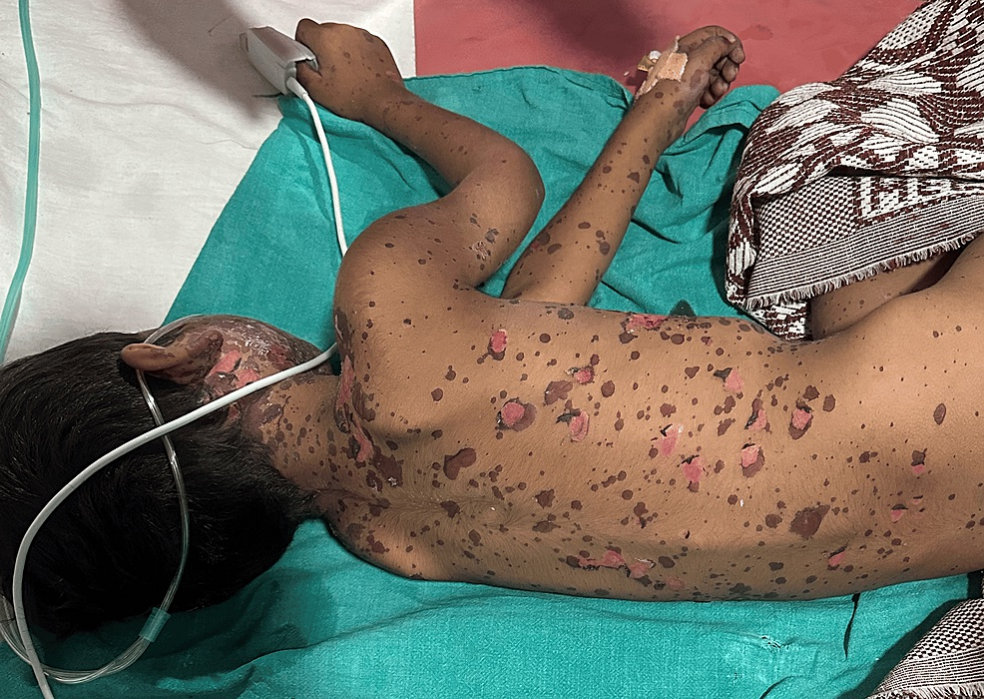
Lesions on the back side of the body

Upon attending the emergency department, it was noted that the patient had multiple purpuric spots generalized over the body on the front side (Figure 1) and the back side (Figure 2).

There was scalp sparing. The lesions exhibited crusting and skin peeling.

There was no significant past or family history of allergic diseases. The patient had achieved all the developmental milestones according to age and was immunized for age. On general examination, the patient was found to be conscious, oriented, and hemodynamically stable. The nutritional status was good. The child was between the 50th and 90th percentile for weight for age and height for age. There were no signs of dehydration. There was no pallor, clubbing, lymphadenopathy, or edema. Laboratory reports for renal and liver function tests were within normal limits.

Based on history and clinical progression, the patient was suspected to have TEN, with the suspected offender being paracetamol. Accordingly, conservative management started with capsules of cyclosporine, tablets of pantoprazole, multivitamin supplements, and intravenous fluids. The skin lesions were cleaned with isotonic saline. Thereafter, hydrogel (colloid gel) dressings were applied for the alleviation of pain and to reduce fluid loss. Skin lesions recovered within seven days of conservative management. There were no complications, and the patient was discharged.

## Discussion

Paracetamol is the most commonly used over-the-counter (OTC) analgesic in India. The warning label includes caution against side effects like drowsiness, tiredness, stomach upset, etc. Caution is to be exercised when prescribing to patients with known gastrointestinal bleeding disorders, breathing difficulties, etc. Serious side effects like Reye’s syndrome involving liver and kidney damage have been reported rarely following excessive and chronic overuse. Hence, the safe daily limit of the drug was defined as 15 mg per kg with a maximum of four doses in 24 hours, especially for pediatric patients [7,8].

Steven Johnson syndrome is a type IV hypersensitivity reaction following exposure to the offending agent. It has been observed that it develops either promptly following exposure or may take up to three weeks [9]. The initial presentation includes an influenza-like picture with generalized skin lesions involving necrosis and sloughing. Mucosal involvement may also be seen. The underlying pathophysiological mechanism involves the activation of cytotoxic T cells following exposure to the offending agent. An increased concentration of these cells causes apoptosis of the keratinocytes, leading to various dermatological manifestations. This leads to increased production of inflammatory cytokines through various signaling pathways, which in turn causes increased production of cytotoxic T cells, leading to a vicious cycle [10,11]. Additionally, annexin A1 from monocytes interacts with the formyl peptide receptor 1 and causes necroptosis [5]. Some drugs are known to trigger this rare immune reaction. These include antiepileptics, oxicams, antibiotics like penicillin, cephalosporins, tetracyclines, etc. [11]. When the area involved is less than 10%, it is called SJS; between 10% and 30% involvement, it is SJS/TEN; and when it is more than 30%, it is termed TEN.

Steven Johnson syndrome/toxic epidermal necrosis following ingestion of paracetamol in recommended doses is rare and hence not considered in the differential diagnosis upon presentation of the patient. Understandably, it leads to a significant delay in starting the appropriate treatment. The first line of management for STS/TEN involves the withdrawal of the offending agent. However, when it is misdiagnosed as a viral infection like varicella zoster, as in the present case, paracetamol is administered as the treatment of the misdiagnosed condition, leading to repeated exposure to the offending agent and possibly worsening of the condition of the patient. Therefore, careful consideration of STS/TEN in the differential diagnosis becomes imperative.

There have been a few reports in Western countries wherein SJS/TEN with paracetamol as the suspected offending agent [12,13]. It has also been observed to be associated with certain specific human leukocyte antigen (HLA) genotypes (HLA-B*13:01, HLA-C*14:03, HLA-A*02:06, and HLA-B*44:03 in the Japanese population and HLA-B*44:03, HLA-A*33:03, and HLA-C*07:01 in the Thai population, particularly in cases associated with severe ocular complications) [14, 15]. However, the underlying pathophysiological mechanism remains to be proven.

## Conclusions

Steven Johnson syndrome/toxic epidermal necrosis following exposure to paracetamol is potentially life-threatening. Awareness amongst physicians regarding STS/TEN is essential not only for early diagnosis and treatment of the condition but also for preventing repeated exposure to the drug, as every potential alternative diagnosis involves paracetamol as the first line of treatment. The use of paracetamol as an OTC analgesic may also be reconsidered and may require administration under strict medical supervision.
